# Supplementary material for: Discovery of Triple Inhibitors of Both SARS-CoV-2 Proteases and Human Cathepsin L
Source: Pharmaceuticals (Basel). 2022 Jun 13;15(6):744. doi: 10.3390/ph15060744 (PMC9230533; doi:10.3390/ph15060744)
Supplement: Supplementary file 1 [file pharmaceuticals-15-00744-s001.zip › pharmaceuticals-1729795-supplementary.pdf]

## Supplementary Data

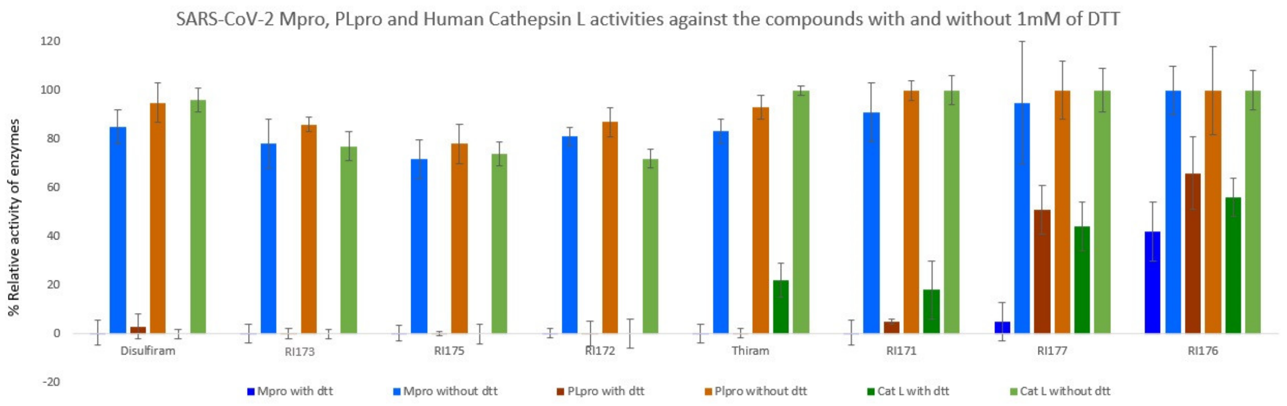


**Figure S1.** SARS-CoV-2 Mpro, PLpro and Human Cathepsin L activities against the compounds with and without 1mM of DTT.

A

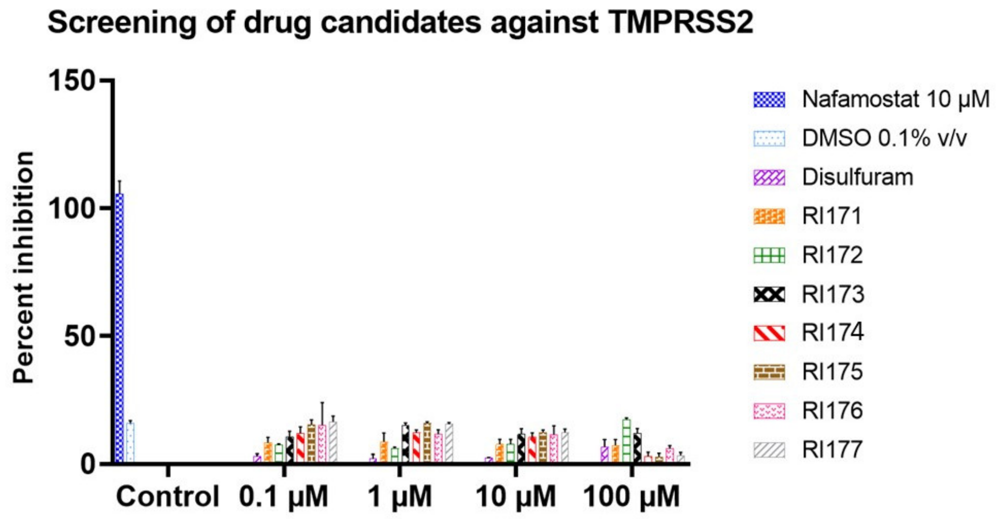


B

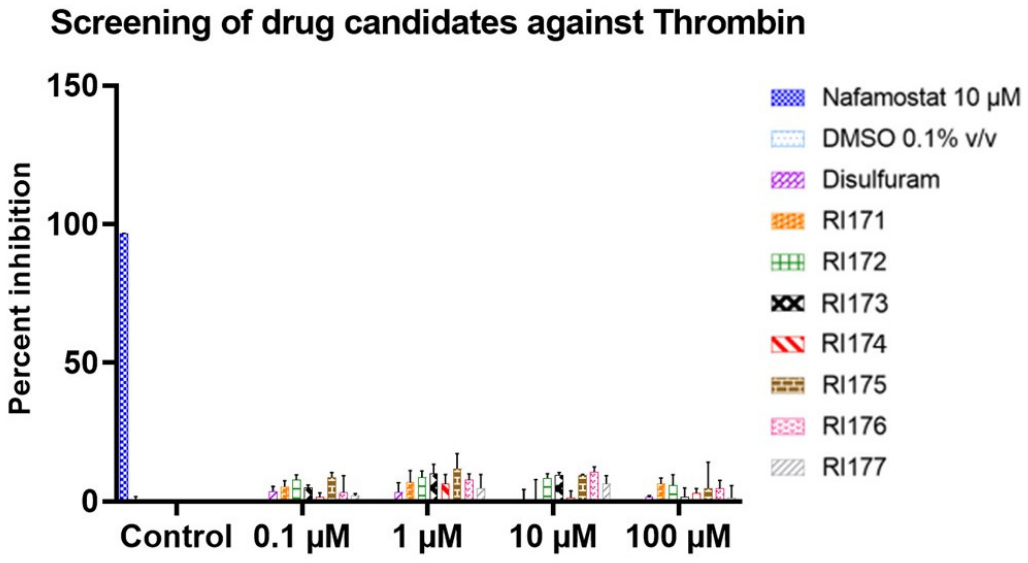


**Figure S2.** Counter-screening on Disulfiram and selected thiuram disulfide compounds at 0.1, 1, 10, 100 µM against 1 nM and 10 nM of (A) TMPRSS2 and (B) Thrombin, respectively.
